# Supplementary material for: Analysis of the impact of RCEP on the industrial and innovation chains of China’s textile and clothing industry
Source: PLoS One. 2024 Aug 30;19(8):e0309708. doi: 10.1371/journal.pone.0309708 (PMC11364412; doi:10.1371/journal.pone.0309708)
Supplement: Attachment — Submitted filename: Response to Reviewers.docx [file pone.0309708.s001.docx]

12^th^ June 2024

Dear Editor and Reviewers,

We want to express our heartfelt gratitude for your invaluable feedback on our manuscript titled " ***Analysis of the Impact of RCEP on the Industrial and Innovation Chains of China's Textile and Clothing Industry***." We have carefully considered your insightful comments and made the necessary revisions. Our updated version has been resubmitted to the Journal of PLOS ONE, and we anticipate your feedback.

We truly appreciate the time and effort you spent reviewing our work. Your constructive suggestions and observations were instrumental in improving the quality and clarity of our research. Your expertise and guidance throughout this process have been invaluable, allowing us to refine and present our work more effectively.

Below, we have included a table outlining our changes to facilitate your review process.

Once again, we extend our sincere appreciation for your time, effort, and invaluable feedback. We eagerly await your response.

Best regards,

Sincerely,

Corresponding Author, P J Kumarasinghe

| **Comments** | **Response** |
| --- | --- |
| 1. Is the manuscript technically sound, and do the data support the conclusions?  The manuscript must describe a technically sound piece of scientific research with data that supports the conclusions. Experiments must have been conducted rigorously, with appropriate controls, replication, and sample sizes. The conclusions must be drawn appropriately based on the data presented.  Reviewer #1: Yes  Reviewer #2: Yes | We appreciate the reviewers' positive feedback, affirming our manuscript's technical soundness and the data supporting the conclusions. We thank you for thoroughly reviewing and validating our research methodology and findings. |
| 2. Has the statistical analysis been performed appropriately and rigorously?  Reviewer #1: Yes  Reviewer #2: Yes | We are grateful for the reviewers' acknowledgment that our statistical analysis has been performed appropriately and rigorously. Thank you for your careful evaluation and positive feedback. |
| 3. Have the authors made all data underlying the findings in their manuscript fully available?  The [PLOS Data policy](http://www.plosone.org/static/policies.action#sharing) requires authors to make all data underlying the findings described in their manuscript fully available without restriction, with rare exception (please refer to the Data Availability Statement in the manuscript PDF file). The data should be provided as part of the manuscript or its supporting information, or deposited to a public repository. For example, in addition to summary statistics, the data points behind means, medians and variance measures should be available. If there are restrictions on publicly sharing data—e.g. participant privacy or use of data from a third party—those must be specified.  Reviewer #1: Yes  Reviewer #2: Yes | We confirm that all data underlying the findings described in our manuscript are fully available without restriction. The data has been deposited in a public repository and can be accessed at the following link:  Kumarasinghe, Pivithuru; Yang, Li (2024), “RCEP on the Industrial and Innovation Chains of China's Textile and Clothing Industry”, Mendeley Data, V1, doi: 10.17632/sgvmg6c9zz.1  Thank you for your attention to this matter. |
| 4. Is the manuscript presented in an intelligible fashion and written in standard English?  PLOS ONE does not copyedit accepted manuscripts, so the language in submitted articles must be clear, correct, and unambiguous. Any typographical or grammatical errors should be corrected at revision, so please note any specific errors here.  Reviewer #1: Yes  Reviewer #2: Yes | We appreciate the reviewers' feedback regarding the clarity and language of our manuscript. We have reviewed the manuscript again to ensure it is presented understandably and written in standard English. |
| Reviewer #1: | |
| 5. Review Comments to the Author  Please use the space provided to explain your answers to the questions above. You may also include additional comments for the author, including concerns about dual publication, research ethics, or publication ethics. (Please upload your review as an attachment if it exceeds 20,000 characters)  Reviewer #1: your study is novel and interesting examining the impact of Regional Comprehensive Economic Partnership (RCEP) on textile and apparel industry. The title, abstract and introduction are fine and valid. The methodology and results are fine too.  you are suggested to use abbreviation with original text at first appearance and then use just the short one later throughout the manuscript. Try to convert some tables to attractive figures to gather attraction frim the readers. Also the figures have some overlapping text with the bars. minor corrections are suggested | We appreciate your positive feedback and constructive suggestions. To address your comments, we have made the following revisions:   1. **Abbreviations**:    - We have ensured that all abbreviations are introduced with their original text at first appearance and then used consistently throughout the manuscript. 2. **Tables and Figures**:    - Tables have been converted into more attractive figures to enhance reader engagement and visual appeal.    - We have corrected the overlapping text in the figures to ensure clarity and readability. |
| Reviewer #2: | |
| Reviewer #2: The article "Analysis of the Impact of RCEP on the Industrial and Innovation Chains of China's Textile and Clothing Industry" considers the impact of Regional Comprehensive Economic Partnership (RCEP) on the textile and clothing industry, trade dynamics, innovation chains, and industrial integration. While there is research on the overall economic effects of RCEP, studies specifically examining its impact on individual industries like textiles are relatively limited. The article "Analysis of the Impact of RCEP on the Industrial and Innovation Chains of China's Textile and Clothing Industry" aims to fill a research gap by analyzing the specific impact of RCEP on innovation chains and industrial integration within the textile sector, going beyond just trade volume changes. Scientific Contribution: - Provides empirical evidence on the impact of RCEP on the textile industry, specifically focusing on innovation and industrial chains. - Offers insights into the potential benefits and challenges of RCEP for the textile sector in China and other member nations. - Contributes to the understanding of how trade agreements can influence innovation and industrial integration in specific industries. I'd recommend for the authors to consider incorporating ESG (Environmental, Social, and Governance) goal achievement into their research: 1. Aligning with Current Trends: ESG considerations are becoming increasingly important for businesses and investors globally. 2. Expanding the Scope: Adding an ESG dimension would broaden the analysis to encompass the social and environmental implications of RCEP on the textile industry. 3. Addressing Sustainability Concerns: Examining how RCEP can contribute to sustainable practices within the industry would be highly valuable. 4. Identifying Opportunities: The research could highlight how RCEP can facilitate the adoption of sustainable practices and create new business models. 5. Policy Recommendations: Incorporating ESG would allow the authors to provide more comprehensive policy recommendations for promoting sustainable growth within the textile sector. | We appreciate the insightful feedback and recommendations. To address these comments, we have revised our manuscript to incorporate ESG considerations to a certain extent. The following sections have been added:  4.7. Integrating ESG Goals into the Analysis of RCEP's Impact on the Textile Industry.  In this section, we discussed the suggested inclusion to a certain extent.  **Refer to Page number(s):21-22**  **Conclusion:**  - Expanded to highlight the integration of ESG goals and their impact on the textile industry under the RCEP framework.  **Refer to Page number(s):23**  **Limitations:**  - Adjusted to acknowledge that while we have incorporated ESG considerations to some extent, time constraints.  **Refer to Page number(s):23**  This remains an area for future research and we are planning to do. Due to time constraints and the complexity of integrating all suggested ESG aspects comprehensively, we have not included some of the detailed suggestions. These limitations have been discussed and acknowledged in the revised limitations section of our manuscript.  Thank you for your valuable feedback, which has significantly enhanced the depth and relevance of our study. |
| 6. PLOS authors have the option to publish the peer review history of their article ([what does this mean?](https://journals.plos.org/plosone/s/editorial-and-peer-review-process#loc-peer-review-history)). If published, this will include your full peer review and any attached files.   If you choose “no”, your identity will remain anonymous but your review may still be made public.   **Do you want your identity to be public for this peer review?** For information about this choice, including consent withdrawal, please see our [Privacy Policy](https://www.plos.org/privacy-policy).  Reviewer #1: No  Reviewer #2: **Yes:** Sergey Barykin | We will not be publishing the peer review history of our article. Therefore, the identity of the reviewers will remain anonymous as per the default policy. |
| **Journal Requirements:** |  |
| 1. Please ensure that your manuscript meets PLOS ONE's style requirements, including those for file naming. The PLOS ONE style templates can be found at  https://journals.plos.org/plosone/s/file?id=wjVg/PLOSOne_formatting_sample_main_body.pdf and  https://journals.plos.org/plosone/s/file?id=ba62/PLOSOne_formatting_sample_title_authors_affiliations.pdf  2. Thank you for stating the following financial disclosure:  "This study received support from the Research project of the Zhejiang Provincial Department of Education (Y202351899) and Zhejiang Federation of Humanities and Social Sciences Circles Base research projects (2014JDZ01)."    Please state what role the funders took in the study.  If the funders had no role, please state: ""The funders had no role in study design, data collection and analysis, decision to publish, or preparation of the manuscript.""  If this statement is not correct you must amend it as needed.  Please include this amended Role of Funder statement in your cover letter; we will change the online submission form on your behalf.  3. Please review your reference list to ensure that it is complete and correct. If you have cited papers that have been retracted, please include the rationale for doing so in the manuscript text, or remove these references and replace them with relevant current references. Any changes to the reference list should be mentioned in the rebuttal letter that accompanies your revised manuscript. If you need to cite a retracted article, indicate the article’s retracted status in the References list and also include a citation and full reference for the retraction notice. | 1. We have reviewed and revised our manuscript to ensure it meets PLOS ONE's style requirements, including those for file naming. We have utilized the provided PLOS ONE style templates to format the main body, title, authors, and affiliations sections accordingly. 2. The statement is correct. "The funders had no role in study design, data collection and analysis, decision to publish, or preparation of the manuscript." We will include this statement in the cover letter. Thank you for updating it in the online submission form on our behalf. 3. We have reviewed our reference list to ensure that it is complete and correct. We have verified that none of the cited papers have been retracted. |
| While revising your submission, please upload your figure files to the Preflight Analysis and Conversion Engine (PACE) digital diagnostic tool, <https://pacev2.apexcovantage.com/>. PACE helps ensure that figures meet PLOS requirements. To use PACE, you must first register as a user. Registration is free. Then, login and navigate to the UPLOAD tab, where you will find detailed instructions on how to use the tool. If you encounter any issues or have any questions when using PACE, please email PLOS at <a href="mailto:figures@plos.org">figures@plos.org. Please note that Supporting Information files do not need this step. | As requested, we have uploaded our figure files to the Preflight Analysis and Conversion Engine (PACE) digital diagnostic tool. All figures have been checked and meet the PLOS requirements. Also, replace the figures with the converted ones using Conversion Engine (PACE). Thank you for providing this tool, which has helped us ensure the quality and compliance of our figures. |

-END-
